# Hyperbaric oxygen therapy modulates immune effector responses and reshapes peripheral immune tolerance: a narrative review

**DOI:** 10.3389/fimmu.2026.1777972

**Published:** 2026-03-03

**Authors:** Shuhao Mei, Boran Dong, Yuling Gao, Jiaqi Zhou, Hailian Yi, Yuyin Han, Wenzhen Zhuo, Mengyan Sun, Meiting Li, Han Wang, Yong Liu, Xiaoyang Gong

**Affiliations:** 1 Department of Rehabilitation Medicine, The First Affiliated Hospital of Dalian Medical University, Dalian, China; 2 College of Health-Preservation and Wellness, Dalian Medical University, Dalian, China

**Keywords:** hyperbaric oxygen therapy, hypoxia-inducible factor signaling, immunometabolism, oxygen-sensing pathway, peripheral immune tolerance, regulatory T cells

## Abstract

Hyperbaric oxygen therapy (HBOT) refers to an intervention in which patients inhale near-100% oxygen at pressures exceeding 1 atmosphere absolute to increase plasma and tissue oxygen partial pressure. HBOT has been applied clinically across a broad range of conditions, including infections, inflammation, hypoxia-related injury, and malignancies. However, its immunological effects are often reduced to a binary notion of “immune enhancement” or “immunosuppression”. Moreover, substantial heterogeneity in treatment parameters and immune endpoints across studies has limited the development of a unified interpretive framework centered on peripheral immune tolerance (PIT). Following the PRISMA 2020 reporting framework, we standardized the presentation of the search and selection process. PubMed, Embase, Web of Science, the Cochrane Library, and Scopus were searched from database inception to November 15, 2025. Two reviewers independently performed study screening and data extraction. Ultimately, 39 relevant articles were included, and a mechanism-oriented qualitative narrative synthesis was conducted along the axes of oxygen tension, immunometabolism, and PIT. Across the included studies, in models of autoimmune and chronic inflammatory disease, HBOT was commonly associated with expansion of regulatory T cells and suppression of T helper 17–related inflammatory pathways, accompanied by a homeostatic recalibration of peripheral tolerance thresholds and improved tissue inflammatory outcomes. Under infectious and hyperinflammatory conditions, pro-inflammatory transcriptional signatures and cytokine responses were attenuated, markers of oxidative damage were reduced, while neutrophil directional bactericidal capacity was enhanced, suggesting synergy with certain antimicrobial therapies. In hypoxic tumor microenvironments, antigen presentation was improved, cytotoxic T-cell infiltration increased, and immunosuppressive myeloid components decreased, collectively indicating potential additive or synergistic benefits with immunotherapy. In summary, we propose an integrated framework in which upstream oxygen tension sensing drives intermediate immunometabolic remodeling, culminating in downstream reprogramming of immune cell lineages and functional states. This framework provides a testable theoretical basis for explaining the context-dependent immunological effects of HBOT across diseases and for guiding prospective study designs incorporating composite immune endpoints and therapeutic windows.

## 1 Introduction

Hyperbaric oxygen therapy (HBOT) is a non-pharmacological intervention in which patients inhale near-100% oxygen at pressures exceeding 1 atmosphere absolute (ATA), most commonly 2.0–3.0 ATA, thereby markedly increasing plasma and tissue oxygen partial pressure (1). The biological effects of HBOT extend well beyond the simple correction of hypoxia; accumulating evidence indicates that HBOT can systemically reshape peripheral immune cell composition and indices related to immune tolerance. Accordingly, peripheral immune tolerance (PIT) has emerged as a central conceptual framework for interpreting the immunological effects of HBOT (2, 3).

PIT refers to a series of mechanisms by which lymphocytes, after exiting the thymus and bone marrow, continuously restrain autoreactive immune responses and limit inflammation. These mechanisms include clonal anergy, clonal deletion, inhibitory co-stimulatory signaling, and active suppression mediated by regulatory T cells (Tregs) (4). Deficiency of CD25^+^/FOXP3^+^ Tregs results in fatal multisystem autoimmunity in both humans with immune dysregulation, polyendocrinopathy, enteropathy, X-linked (IPEX) syndrome and scurfy mice, whereas ectopic acquisition of FOXP3 expression is sufficient to confer suppressive function on conventional T cells. Together, genetic and functional evidence firmly establishes FOXP3^+^ Tregs as a core cellular determinant of PIT (5). Recent advances, including single-cell suppression profiling, have further delineated microenvironment-specific suppressive programs of human Tregs. In parallel, targeted strategies exemplified by manipulation of the CD2–LFA-3 axis can selectively inhibit high-CD2-expressing effector T cells while relatively sparing Tregs, thereby restoring immune homeostasis across multiple autoimmune models (6, 7). Within this shared regulatory network, T helper 17 (Th17) cells and Tregs display distinct metabolic dependencies: Th17 cells preferentially rely on glycolysis and amino-acid metabolism, whereas Tregs are biased toward fatty-acid oxidation and mitochondrial oxidative phosphorylation (OXPHOS). Mitochondria-derived reactive oxygen species (ROS), mitochondrial dynamics, and processes associated with immune aging and inflammaging jointly shape the Th17/Treg balance and overall T-cell functional states (8, 9). By enabling precise, whole-body modulation of oxygen tension and inducing controlled mitochondrial responses, HBOT can directly engage the oxygen tension–immunometabolism–PIT axis (10).

PIT is often described in terms of the Treg/Th17 axis. However, tolerance induction and maintenance involve multiple immune compartments (4, 11). Innate immune cells, including neutrophils, monocytes/macrophages, and dendritic cells, shape early inflammatory thresholds, regulate antigen presentation, and define cytokine environments that steer subsequent T-cell polarization (12, 13). Natural killer (NK) cells, γδ T cells, and CD8^+^ T cells may also contribute via rapid effector functions and oxygen-sensing or immunometabolic pathways (14). Evidence from acute injury and toxic exposure models can help characterize upstream immune changes reported under HBOT and inform mechanistic links to tolerance-associated alterations described in chronic disease settings.

Despite the growing number of studies reporting immunological effects of HBOT, substantial heterogeneity persists with respect to disease models, treatment protocols, and endpoint selection. Most investigations emphasize anti-inflammatory, anti-infective, or tissue-repair outcomes, and a unifying interpretative perspective centered on PIT remains largely absent. To enhance transparency in evidence retrieval and appraisal, the present study adheres to the Preferred Reporting Items for Systematic Reviews and Meta-Analyses (PRISMA) 2020 reporting framework (15) and adopts PIT as the core analytical lens. We therefore conduct a mechanism-oriented qualitative narrative synthesis and critical analysis of existing evidence, focusing on how HBOT reshapes PIT within a conceptual framework integrating FOXP3^+^ Tregs and oxygen tension–immunometabolic coupling. From a methodological standpoint, this work constitutes a mechanistic narrative review grounded in systematic literature retrieval.

## 2 Methods

### 2.1 Literature search strategy

The literature search and screening procedures were conducted and reported in accordance with the PRISMA 2020 statement (15), with the aim of standardizing the description of data sources, screening steps, and inclusion/exclusion criteria. On this basis, a mechanism-oriented narrative review grounded in systematic retrieval was performed, integrating available evidence through qualitative narrative synthesis and critical analysis. A comprehensive and systematic search was carried out in the major bibliographic databases PubMed, Embase, Web of Science, Cochrane Library, and Scopus, covering the period from database inception to November 15, 2025. The search strategy combined controlled vocabulary terms and free-text keywords. Broad immunology-related terms, such as “immunity” and “immune*,” were deliberately incorporated into the search strings to enhance sensitivity and minimize the risk of missing relevant studies due to heterogeneity in terminology. This approach was intended to capture HBOT studies reporting peripheral immune modulation outcomes even when immune tolerance or Treg-related terms were not explicitly stated in the title or abstract. The detailed search strategy and Boolean logic are presented in **Table 1**, using the PubMed search syntax as a representative example.

**Table 1:** Database search strategy.

Number	Search strategy
#1	“Immune Tolerance”[Mesh]
#2	(“Immune Tolerance”[Title/Abstract]) OR (“Immunosuppression*”[Title/Abstract]) OR (“Tolerance, Immune”[Title/Abstract])
#3	#1 OR #2
#4	“T-Lymphocytes, Regulatory”[Mesh]
#5	(“Regulatory T Cell*”[Title/Abstract]) OR (“Cell*, Regulatory T”[Title/Abstract]) OR (“T Cell*, Regulatory”[Title/Abstract]) OR (“Regulatory T-Lymphocyte*”[Title/Abstract]) OR (“Regulatory T Lymphocyte*”[Title/Abstract]) OR (“T-Lymphocyte*, Regulatory”[Title/Abstract]) OR (“Treg Cell*”[Title/Abstract]) OR (“Cell*, Treg”[Title/Abstract]) OR (“Regulatory T-Cell*”[Title/Abstract]) OR (“T-Cell*, Regulatory”[Title/Abstract]) OR (“Tr1 Cell*”[Title/Abstract]) OR (“Cell*, Tr1”[Title/Abstract]) OR (“Cell*, Th3”[Title/Abstract]) OR (“Th3 Cell*”[Title/Abstract]) OR (“Naturally-Occurring Suppressor T-Lymphocyte*”[Title/Abstract]) OR (“Suppressor T-Lymphocyte*, Naturally-Occurring”[Title/Abstract]) OR (“Suppressor T Lymphocyte*, Naturally Occurring”[Title/Abstract]) OR (“Suppressor T-Cell*, Naturally-Occurring”[Title/Abstract]) OR (“Naturally-Occurring Suppressor T-Cell*”[Title/Abstract]) OR (“Suppressor T Cell*, Naturally Occurring”[Title/Abstract]) OR (“T-Cell*, Naturally-Occurring Suppressor”[Title/Abstract])
#6	#4 OR #5
#7	“Immunity”[Mesh]
#8	(“Immunity*”[Title/Abstract]) OR (“Immune*”[Title/Abstract])
#9	#7 OR #8
#10	#3 OR #6 OR #9
#11	“Oxygen”[Mesh]
#12	(“Oxygen”[Title/Abstract]) OR (“Oxygen-16”[Title/Abstract]) OR (“Oxygen 16”[Title/Abstract]) OR (“Dioxygen”[Title/Abstract])
#13	#11 OR #12
#14	“Reactive Oxygen Species”[Mesh]
#15	(“Reactive Oxygen Species”[Title/Abstract]) OR (“Oxygen Radical*”[Title/Abstract]) OR (“Radical, Oxygen”[Title/Abstract]) OR (“Reactive Oxygen Intermediates”[Title/Abstract]) OR (“Oxygen Species, Reactive”[Title/Abstract]) OR (“Pro-Oxidant*”[Title/Abstract]) OR (“Pro Oxidant*”[Title/Abstract]) OR (“Active Oxygen*”[Title/Abstract]) OR (“Oxygen, Active”[Title/Abstract])
#16	#14 OR #15
#17	#13 OR #16
#18	“Hyperbaric Oxygenation”[Mesh]
#19	(“Hyperbaric Oxygenation*”[Title/Abstract]) OR (“Oxygenation*, Hyperbaric”[Title/Abstract]) OR (“Hyperbaric Oxygen Therap*”[Title/Abstract]) OR (“Oxygen Therap*, Hyperbaric”[Title/Abstract]) OR (“Therap*, Hyperbaric Oxygen”[Title/Abstract])
#20	#18 OR #19
#21	#10 AND #17 AND #20

The full Boolean query is shown for PubMed. For Embase, Web of Science, Cochrane Library, and Scopus, the strategy was adapted using database-specific field tags and syntax while retaining the same core concepts.

### 2.2 Inclusion and exclusion criteria

#### 2.2.1 Inclusion criteria

A structured inclusion framework was applied in this study. For interventional studies, the Population, Intervention, Comparison, Outcomes, and Study design (PICOS) principle was used as the primary guide, supplemented by criteria based on mechanistic relevance (15). The objective was to integrate evidence linking HBOT to PIT from a mechanistic perspective rather than to quantitatively pool intervention effects. Accordingly, peer-reviewed publications of diverse study designs were eligible for inclusion provided that they met all of the following criteria: (1) Intervention: Exposure to HBOT at pressures ≥ 1.4 ATA, either as a standalone intervention or as an adjunctive therapy. Conventional clinical HBOT protocols typically employ pressures ≥ 2.0 ATA (16). To comprehensively capture evidence related to mild HBOT, the lower pressure threshold was set at 1.4 ATA (17). The single study using a 1.4 ATA protocol was analyzed separately in the Results and Discussion sections with respect to its potential mechanistic differences and limitations. (2) Outcomes: Explicit reporting of endpoints related to PIT or immune modulation, including but not limited to peripheral blood lymphocyte subsets, functional or quantitative measures of myeloid cells, systemic cytokine profiles, and biomarkers associated with immune tolerance or immunosenescence. (3) Study types: *In vitro* experiments, animal studies, clinical investigations, modeling studies, and reviews addressing key mechanisms linking HBOT and PIT. Review articles were included solely to supplement mechanistic insights and contextual background; they were not used as direct evidence for effect direction or strength, and duplication of primary studies covered by these reviews was avoided.

#### 2.2.2 Exclusion criteria

To ensure the specificity and reliability of the analysis, studies meeting any of the following criteria were excluded: (1) Ineligible interventions: Studies involving normobaric oxygen therapy only, or oxygen exposures that did not meet the pressure requirements for HBOT; (2) Limited outcomes: Studies focusing exclusively on local histopathological changes or isolated oxygenation parameters, without providing readouts that were informative for HBOT-mediated PIT mechanisms; (3) Publication type: Conference abstracts, case reports, editorials, books, or short communications for which complete datasets were unavailable; (4) Data redundancy: Duplicate publications derived from the same dataset, or records that, upon evaluation, failed to provide additional mechanistic contributions beyond those of already included studies.

### 2.3 Study selection process

**Figure 1:**
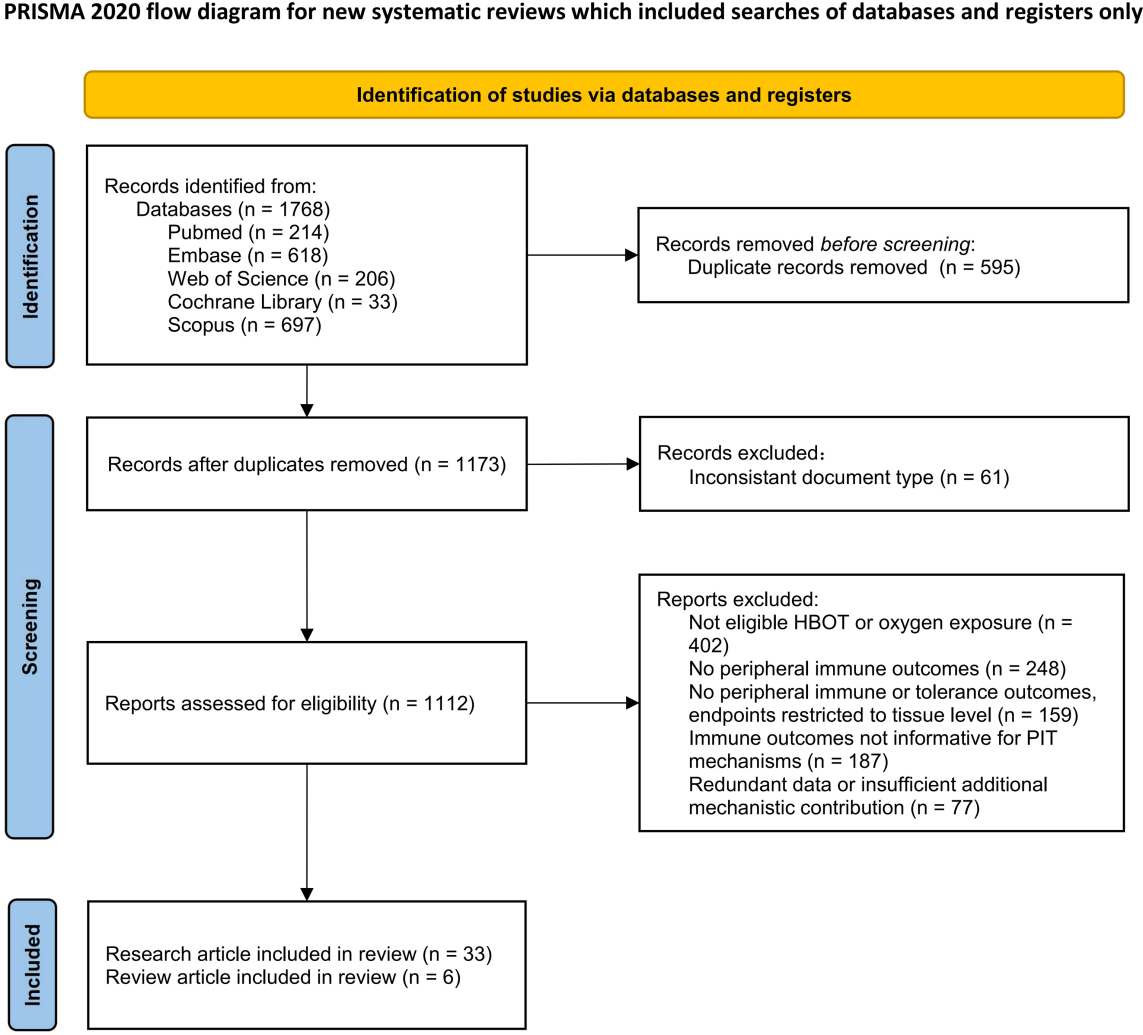
Flow diagram of literature identification and screening.

The study selection process was organized and reported in accordance with the flow diagram recommended by the PRISMA 2020 statement to enhance transparency and traceability of the search and inclusion/exclusion procedures (15). After removal of duplicate records, two investigators independently screened titles and abstracts to exclude clearly irrelevant studies. Full texts of potentially eligible articles were then retrieved and assessed against the predefined inclusion and exclusion criteria to determine final eligibility. Any discrepancies during the screening process were resolved through discussion or, when necessary, consultation with a third investigator. A total of 1,768 records were initially identified, and following the standardized screening workflow, 39 studies were ultimately included as the evidentiary basis of this review. The detailed selection process is illustrated in **Figure 1**.

Flow diagram of literature identification, screening and inclusion in this review, in accordance with the Preferred Reporting Items for Systematic Reviews and Meta-Analyses (PRISMA) 2020 reporting. A total of 1,768 records were retrieved from five databases.

### 2.4 Data extraction and qualitative synthesis

Systematic data extraction was performed for all 39 studies included in this review. Extracted variables comprised the first author and year of publication, the disease model and study population, detailed HBOT intervention parameters—including pressure level, duration of each exposure, treatment frequency, and total treatment course—as well as key peripheral immune endpoints. Data extraction was conducted independently by two investigators. Any discrepancies were resolved through discussion or, when necessary, adjudication by a third investigator, thereby ensuring consistency and completeness of the extracted information.

Given the substantial heterogeneity across included studies with respect to study design, disease context, intervention parameters, and outcome assessment, a qualitative narrative synthesis approach was adopted (18). Specifically, a series of analytical themes was constructed based on immune cell lineages and signaling pathways. *In vitro* experiments, animal studies, clinical investigations, modeling studies, and relevant mechanistic reviews were grouped under the corresponding thematic categories. Within each theme, consistency and divergence of evidence across studies were comparatively evaluated and subsequently synthesized in narrative form. This strategy provided the foundation for an integrated interpretation of the regulatory patterns and mechanistic pathways through which HBOT influences PIT.

## 3 Overview of included studies

A total of 39 peer-reviewed publications were included in this review. The majority were original research articles (n = 33, 84.6%), comprising 16 animal studies, 5 *in vitro* or ex vivo studies, 11 clinical studies, and 1 Bayesian modeling study. Six review articles (15.4%) were also included, predominantly narrative reviews, with one systematic review. Among the original studies, animal experiments largely focused on two major contexts: autoimmune and chronic inflammatory conditions, aimed at examining changes in organ-specific and systemic immune responses; and acute injury or toxic exposure models, emphasizing acute tissue damage and secondary inflammatory processes. *In vitro* and ex vivo studies typically employed peripheral immune cells, pathogen-related systems, or isolated tissues to evaluate the effects of HBOT on antimicrobial efficacy and potential synergistic interactions, while also assessing stress responses and immunogenicity-related endpoints. Clinical studies spanned a wide range of disease settings, including acute severe infections and toxic encephalopathy, as well as chronic autoimmune or inflammatory disorders. These investigations further extended to populations characterized by immune reconstitution or immune activation states, with healthy volunteers and individuals with mild to moderate chronic inflammation serving as reference groups. The Bayesian modeling study integrated existing experimental and clinical data to jointly model and predict HBOT-associated immune parameters, thereby providing a systems-level perspective on the interaction between HBOT and PIT. Collectively, these diverse disease models and experimental approaches provided a relatively comprehensive evidence base for the subsequent narrative synthesis and mechanistic integration.

Across the studies included in this review, HBOT intervention pressures were predominantly in the range of 2.0–2.8 ATA (38/39, 97.4%), with single-session exposure durations of 60–120 min. Treatment frequency was most commonly once daily, and total treatment courses ranged from a single exposure to up to 4 consecutive weeks. Only one study (19) employed a mild HBOT protocol (1.4 ATA, 70 min, single session) to examine peripheral NK cell function in healthy women. As the sole low-pressure intervention included in this review, this study provides an important reference point for exploring pressure thresholds and mechanistic specificity underlying HBOT-induced immune effects. The six review articles primarily summarized the potential roles of conventional clinical HBOT protocols (2.0–2.8 ATA) in sepsis, tumor immunity, and transplant immunology. Detailed data extracted from the included studies are presented in **Table 2**.

**Table 2:** Characteristics and key immune outcomes of included studies.

Study	Model/Subject	HBOT protocol	Key metrics	Outcome
I. Clinical studies				
Kjellberg et al., 2024 (48)	COVID-19 ARDS	2.4 ATA, 90–120 min, Daily × 10–20 d	Transcriptomics, PaO_2_	Hypoxemia ↓; Inflammatory gene signatures ↓; Clinical recovery ↑
Vinkel et al., 2023a (45)	NSTI Patients	2.8 ATA, 90 min, Day 1: 3 sessions, then Daily × 5–10 d	Gene expression	Pro-inflammatory gene expression ↓; Metabolic pathway homeostasis ↑
Shroff et al., 2023 (28)	SLE Patients	2.0 ATA, 90 min, Daily × 10 d	Retinal anatomy, Vision	Macular edema ↓; Visual acuity ↑
Nisa et al., 2023 (19)	Healthy Women	1.4 ATA, 70 min, Single session (1 d)	NK cell count and activity	NK cell number ↑; NK cytolytic activity ↑; Systemic inflammation =
de Wolde et al., 2022 (38)	Healthy Volunteers	2.4 ATA, 90 min, Single session (1 d)	ROS, Cytokines	ROS ↑ (Transient); Antioxidant capacity ↑; Systemic cytokines =
Hedetoft et al., 2021a (46)	NSTI Patients	2.8 ATA, 90 min, Day 1: 3 sessions, then Daily × 5–10 d	Plasma Cytokines	IL-6 ↓; IL-8 ↓; G-CSF ↓ (Acute phase response analyzed)
Hedetoft et al., 2021b (46)	NSTI Patients	2.8 ATA, 90 min, ≥3 sessions; 2 sessions within first 24 h preferred	Oxidative stress	MPO ↑, SOD ↑, HO-1 ↑, NOx (nitrite + nitrate) =
Zhang et al., 2021 (61)	CO Poisoning (DE)	2.0 ATA, 60 min, Daily × 20 d	CD34^+^ Stem cells, BDNF	Circulating CD34^+^ cells ↑; BDNF ↑; Delayed encephalopathy ↓
Tillmans et al., 2019 (39)	Healthy Divers	2.8 ATA, 60 min, Single dive exposure (1 d)	Immune subsets	Immune phenotypes =; Pathological inflammation = (Safety confirmed)
Rinaldi et al., 2011 (47)	MOF Patients	2.5 ATA, 90 min, Twice daily × 4 d	TLR pathway, NF-κB	TLR2/4 expression ↓; NF-κB activity ↓; Organ function ↑
Schnittger et al., 2004 (50)	CO Poisoning	2.0 ATA, 120 min, Single session (1 d)	CD18, Lipid peroxidation	Neutrophil CD18 expression ↓; Lipid peroxidation ↓; Hyperactivation ↓
II. *In vivo* animal studies				
a. Mouse models				
Liu et al., 2025 (40)	Mice (SCI)	2.0 ATA, 60 min, Daily × 7 d	γδ T cells, IL-17A	IL-17A^+^ γδ T cells ↓; Neutrophil infiltration ↓; BMS score ↑
Yuen et al., 2024 (55)	Mice (Glioblastoma)	2.5 ATA, 90 min, Daily × 18 d	Macrophage M1/M2	M2 type (CD206^+^) ↓; M1 type (CD86^+^) ↑; TME remodeling ↑
Xiao et al., 2022 (30)	Mice (Tumor 4T1)	2.0 ATA, 60 min, Daily × 14 d	DC maturation, CD8^+^ T	DC maturation ↑; CTL infiltration ↑; Tumor volume ↓↓↓
Liu et al., 2021 (31)	Mice (Solid Tumor)	2.0 ATA, 60 min, Daily × 12 d	PD-1 delivery, MDSC	PD-1 mAb delivery ↑; CD8^+^ T infiltration ↑; MDSC ↓; M2 macrophages ↓
Novak et al., 2016 (57)	Mice (Colitis)	2.0 ATA, 60 min, Twice daily × 7 d	HIF-1α, Cytokines	Colonic HIF-1α mRNA ↓; IL-1β and IL-6 ↓; SOD/CAT activity ↑
Drevet et al., 2014 (43)	Mice (Dermatitis)	2.5 ATA, 60 min, Daily × 14 d	Skin ROS, Th2 cytokines	Skin ROS ↑; IL-4 and IL-5 ↓; IgE levels ↓; Dermatitis score ↓
Faleo et al., 2012 (21)	Mice (NOD Diabetes)	2.0 ATA, 60 min, 5 days/week × 4 w	β-cells, Foxp3^+^ Treg	β-cell proliferation ↑; Foxp3^+^ Treg ↑; Insulitis ↓; Diabetes incidence ↓
Kudchodkar et al., 2008 (54)	Mice (Atherosclerosis)	2.4 ATA, 90 min, Daily × 35 d	Macrophages, Plaque	Aortic M1 macrophages ↓; TNF-α/IL-6 ↓; Plaque area ↓
Chen et al., 2003 (32)	Mice (Lupus)	2.5 ATA, 90 min, Daily × 14 d	Anti-dsDNA, Proteinuria	Anti-dsDNA titers ↓; Proteinuria onset ↓; Splenic IFN-γ/IL-17 ↓
MacKenzie et al., 2000 (22)	Mice (Skin Allograft)	2.5 ATA, 90 min, Daily × 10–14 d	Graft survival, CD4^+^ T	Graft survival ↑ (Tolerance); Tolerance with CD4^+^ depletion = (Lost)
b. Rat models				
Chen et al., 2025 (59)	Rats (CO Lung)	2.5 ATA, 60 min, Single session (1 d)	Mitophagy (Pink1)	Pink1/Parkin mitophagy ↓; Mitochondrial dynamics ↑; Lung injury ↓
Nyam et al., 2024 (41)	Rats (TBI)	2.0 ATA, 60 min, Daily × 7 d	Gut microbiota, TNF-α	*Prevotella copri* abundance ↑; Brain TNF-α ↓; Neuroinflammation ↓
Moon et al., 2017 (20)	Rats (Arthritis)	2.4 ATA, 60 min, Daily × 14 d	Treg/Th17 balance	Splenic Treg ↑; Th17 cells ↓; Serum IL-6 ↓; Joint destruction ↓
Qian et al., 2017 (58)	Rats (TBI)	2.0 ATA, 60 min, Daily × 5 d	NLRP3 inflammasome	NLRP3/ASC/Caspase-1 ↓; IL-1β/IL-18 ↓; Neuronal apoptosis ↓
Bai et al., 2013 (42)	Rats (Pancreatitis)	2.0 ATA, 60 min, Twice daily × 3 d	Lymphocyte apoptosis	Peripheral lymphocyte apoptosis ↑; CD4/CD8 ratio =; Pancreatic necrosis ↓
Thom et al., 2006 (51)	Rats (CO Poisoning)	2.8 ATA, 45 min, Single session (1 d)	Leukocyte sequestration	ICAM-1 dependent leukocyte sequestration ↓; MBP oxidation ↓
III. *In vitro* and ex vivo studies				
Schwartz et al., 2021 (52)	Neutrophils	2.0 ATA, 60 min, Single exposure (1 d)	Bactericidal function	*S. aureus* killing ↑; Cell viability =; Antibiotic synergy ↑
Budiarti et al., 2020 (44)	PBMC (HIV)	2.4 ATA, 90 min, Daily × 5 d	Viral replication (p24)	HIV viral replication ↓; p24 antigen levels ↓
Møller et al., 2019 (53)	*P. aeruginosa*	2.8 ATA, 90 min, Single exposure (1d)	Biofilm, Antibiotics	Biofilm bacterial survival ↓; O_2_ penetration ↑; Ciprofloxacin efficacy ↑
Witte et al., 2014 (60)	Human PBMC	2.5 ATA, 60 min, Single exposure (1 d)	DNA damage (Comet)	DNA single-strand breaks ↑ (Dose-dependent); Cell viability =
MacKenzie et al., 2003 (24)	Fetal Pancreas	2.5 ATA, 90 min, Daily × 14 d	HLA-DR, Immunogenicity	HLA-DR expression ↓; Lymphocyte proliferative response ↓; Graft survival ↑
IV. Bayesian modelling study				
Jermakow et al., 2025 (33)	Bayesian Model	2.0–2.5 ATA, 90 min, Daily × 7 d	Cytokine storm (IL-6)	IL-6 peak intensity ↓; Treg expansion rate ↑; Storm duration ↓
V. Review articles				
Wang et al., 2023 (29)	Review (Tumor)	2.0–2.5 ATA, 60–90 min, Daily × 10–20 d	Hypoxia, Immune surveillance	Tumor hypoxia ↓; Immunotherapy sensitivity ↑; Immunosuppression ↓
Vinkel et al., 2023b (45)	Review (Sepsis)	2.8 ATA, 90 min, Day 1: 3 sessions, then Daily × 7 d	Host response	Systemic inflammatory storm ↓; Host homeostasis ↑
Alenazi et al., 2021 (26)	Review (IBD)	1.5–3 ATA, 5 days/week, 10–40 total sessions	Mucosal healing	Intestinal inflammation ↓; Mucosal repair ↑
Rossignol et al., 2012 (36)	Systematic Review	2.0–2.5 ATA, 90 min, Daily × 20–40 d	Clinical response rate	Clinical remission ↑; Fistula healing ↑; CRP/ESR ↓
Calzia et al., 2006 (56)	Review (Sepsis)	2.0–2.8 ATA, 60–90 min, Twice daily × 3–7 d	Oxygen delivery	Neutrophil adhesion = or ↓; Tissue oxygenation ↑
Al-Waili et al., 2006 (56)	Review (Tx)	2.0–2.5 ATA, 90 min, Daily × 14 d	Lymphoid function	Graft survival ↑; Lymphoid system inflammation ↓

Studies (n = 39) are grouped by study design. The model/subject, hyperbaric oxygen therapy (HBOT) regimen (pressure, session duration, frequency, and total course), the main reported changes in peripheral immune readouts, and associated mechanistic clues are summarized.

Abbreviations: ARDS: Acute respiratory distress syndrome; ASC: Apoptosis-associated speck-like protein containing a CARD (adaptor protein); ATA: Atmospheres absolute; BDNF: Brain-derived neurotrophic factor; BMS: Basso Mouse Scale; CAT: Catalase; CO: Carbon monoxide; CRP: C-reactive protein; CTL: Cytotoxic T lymphocytes; DC: Dendritic cells; DE: Delayed encephalopathy; ESR: Erythrocyte sedimentation rate; G-CSF: Granulocyte-colony stimulating factor; HBOT: Hyperbaric oxygen therapy; HIF-1α: Hypoxia-inducible factor 1-alpha; HIV: Human immunodeficiency virus; HLA-DR: Human Leukocyte Antigen–DR isotype; HO-1: Heme oxygenase-1; IBD: Inflammatory bowel disease; ICAM-1: Intercellular adhesion molecule 1; IgE: Immunoglobulin E; LOOH: Lipid hydroperoxides; MBP: Myelin basic protein; mAb: Monoclonal antibody; MDA: Malondialdehyde; MDSC: Myeloid-derived suppressor cells; MOF: Multiple organ failure; MPO: Myeloperoxidase; NF-κB: Nuclear factor kappa B; NLRP3: NLR family pyrin domain containing 3; NOx: nitric oxide metabolites; NSTI: Necrotizing soft tissue infection; PaO_2_: Partial pressure of arterial oxygen; PBMC: Peripheral blood mononuclear cells; PD-1: Programmed cell death protein 1; SCI: Spinal cord injury; SLE: Systemic lupus erythematosus; SOD: Superoxide dismutase; TBI: Traumatic brain injury; TLR: Toll-like receptor; TME: Tumor microenvironment; Tx: Transplantation; d: day(s); w: week(s); ↑: Increased; ↓: Decreased; =: No significant change.

## 4 Treg/Th17 axis–oriented shift toward immune tolerance

Animal and transplantation-related studies provide relatively direct preclinical evidence that HBOT promotes an immune-tolerant phenotype. Across canonical models of autoimmunity and inflammation, HBOT increases the frequency and suppressive function of FOXP3^+^ Tregs while inhibiting Th17 differentiation and reducing Th17-associated cytokines, including IL-17A, IL-22, and IL-23. Together, these effects lower the Th17/Treg ratio. This pattern has been repeatedly observed in autoimmune models. In collagen-induced arthritis in rats, HBOT increased splenic Treg frequencies and decreased Th17 proportions, coinciding with reduced synovial inflammatory infiltration and improved bone destruction scores (20). In non-obese diabetic (NOD) mice, HBOT delayed diabetes onset and preserved residual pancreatic β-cell mass; both peripheral blood and islet microenvironments showed reduced Th17-associated inflammatory mediators and increased Treg markers (21).

Transplantation-oriented evidence further supports a Treg-dependent shift toward tolerance. In murine skin transplantation, HBOT treatment of recipients enabled long-term graft acceptance, whereas selective depletion of CD4^+^CD25^+^ Tregs rapidly abolished immune unresponsiveness (22). This rapid loss of tolerance after Treg depletion highlights the importance of Treg-mediated active suppression in HBOT-associated tolerance induction. Consistently, transplant immunology reviews have proposed that HBOT may facilitate transplant tolerance by modulating lymphatic inflammation and broader immune regulatory processes (23). In addition, human fetal islet allotransplantation studies indicate that HBOT preconditioning downregulates HLA class II expression in donor tissue, attenuates recipient T-cell proliferative responses, and prolongs graft survival (24).

**Figure 2:**
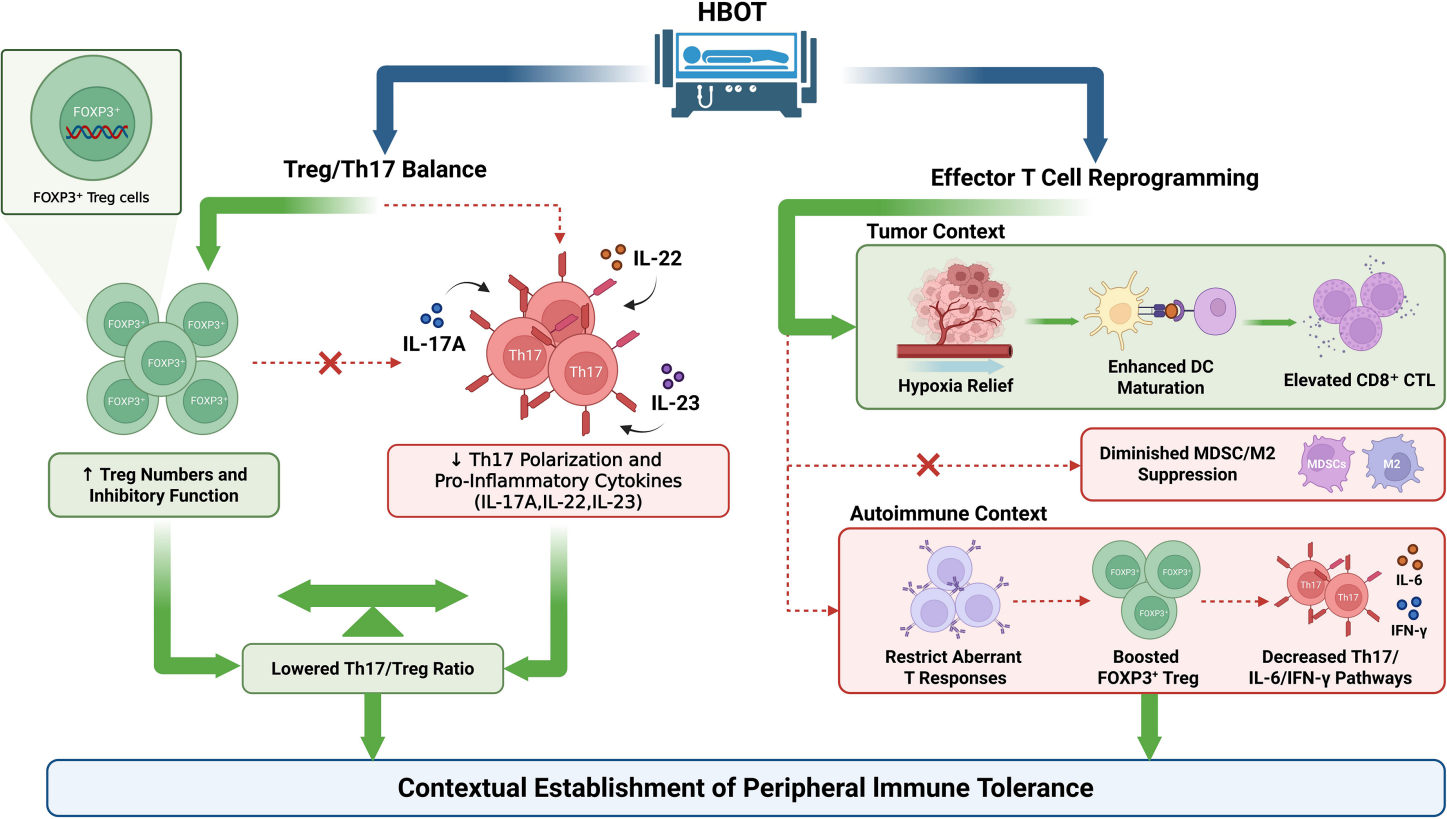
Hyperbaric oxygen therapy (HBOT)–associated T-cell remodeling promotes peripheral immune tolerance (PIT).

Clinical evidence remains limited but is broadly concordant with preclinical findings. In inflammatory bowel disease, HBOT combined with standard therapy improved mucosal healing and induced clinical remission (25, 26), accompanied by immunoregulatory changes consistent with increased Treg activity and reduced pro-inflammatory cytokine signaling, including IL-6 and IL-17–associated pathways (27). Collectively, these data support the conclusion that a central cellular effect of HBOT in PIT remodeling involves the selective enhancement of FOXP3^+^ Treg abundance and function alongside suppression of Th17 polarization and its downstream pro-inflammatory pathways. This dual modulation reduces the Th17/Treg ratio and favors a tolerance-promoting immune microenvironment. However, most clinical studies prioritize disease outcomes as primary endpoints, and direct immunological readouts of the Treg/Th17 axis remain scarce. For example, in systemic lupus erythematosus (SLE)-associated acute macular neuroretinopathy (AMN) cases, the addition of HBOT to immunosuppressive therapy not only significantly improved tissue oxygenation but also exhibited immunomodulatory effects, consistent with a tolerogenic shift toward increased Treg and reduced Th17 responses (28). Therefore, these mechanistic inferences require confirmation and quantification in prospective clinical studies that prespecify immune tolerance axes as endpoints (**Figure 2**).

This conceptual schematic summarizes how HBOT may reshape PIT through T-cell centered remodeling in a context dependent manner, contrasting autoimmune and chronic inflammatory settings with tumors and chronic infections.

Symbol legend: Arrows (→) denote directional functional/regulatory relationships within the proposed framework and do not represent physical trafficking of cells, molecules, or signals. Thick blue solid arrows indicate HBOT as the upstream input/overall driver. Green solid arrows indicate positive regulation, and green bidirectional arrows indicate bidirectional shifts/remodeling of immune balance. Red dashed arrows indicate negative regulation, whereas red dashed arrows labeled with “×” indicate pronounced suppression/blockade of the indicated pathway or amplification loop. Small black arrows indicate within-module process flow or stepwise connections; the direction of regulation is defined by the green/red arrows. Context partitions highlight context-dependent immune outcomes.

## 5 Context-dependent reprogramming of effector T cell lineages and synergy with PIT

In the context where the Treg/Th17 axis has been shifted toward tolerance by HBOT, the reprogramming of effector T cell lineages determines whether peripheral immunity favors immune surveillance or progresses to tissue-damaging inflammation. Preclinical tumor models and systematic reviews focused on tumor immunity jointly indicate that HBOT, by alleviating tumor microenvironment hypoxia, significantly optimizes the metabolic conditions and delivery of effector T cells within the local tumor site (29). In solid tumor mouse models, HBOT at approximately 2.0 ATA increased dendritic cell maturation, enhanced antigen-presenting capacity, boosted CD8^+^ cytotoxic T cell infiltration, and markedly reduced tumor volume (30). Further experiments revealed that HBOT also enhances the delivery of PD-1 monoclonal antibodies within tumor tissues, increasing CD8^+^ T cell numbers while significantly reducing myeloid-derived suppressor cells (MDSC) and M2 macrophages, thereby decreasing tumor immune suppression and increasing the sensitivity to immunotherapy (31). Taken together, these findings suggest that HBOT not only maintains baseline tolerance to self-antigens but also restores effective surveillance and clearance of malignant clones in the tumor setting by relieving hypoxia, improving PD-1 blockade drug delivery, and reshaping the balance between cytotoxic T lymphocytes (CTLs) and myeloid cells.

In autoimmune and organ-specific inflammatory models, by contrast, HBOT preferentially supports FOXP3-mediated regulatory processes. By expanding the Treg pool and improving local cellular stress states, HBOT helps create an environment in which β cells are more likely to be tolerated rather than attacked. In a lupus model, HBOT reduced anti-dsDNA antibody titers, delayed the onset of proteinuria, and ameliorated immune-complex deposition (32). Studies in arthritis rats further suggested that HBOT increases splenic Treg levels, decreases Th17 cell proportions, and reduces serum IL-6, resulting in relief from joint destruction and synovial inflammation (20). In transplant studies, preconditioning with HBOT at approximately 2.5 ATA decreased Human Leukocyte Antigen-DR isotype (HLA-DR) expression in fetal pancreatic tissue, reduced recipient lymphocyte proliferation, and extended graft survival (24). Skin graft experiments further demonstrated that HBOT prolonged graft survival and induced long-term acceptance. However, selective depletion of CD4^+^CD25^+^ Tregs rapidly reversed this tolerant phenotype, underscoring the critical role of Treg-mediated active suppression in HBOT-induced transplant tolerance (22).

Bayesian modeling and clinical reviews provide systemic-level support for these cellular observations. A Bayesian model showed that HBOT significantly reduces the peak intensity of IL-6 during cytokine storms, shortens the duration of high-inflammatory periods, and enhances Treg expansion rates (33). Systematic reviews report that adjunctive HBOT added to standard therapy is associated with improved clinical outcomes in refractory fistulizing perianal and perineal Crohn’s disease, and clinical remission has been observed in some patients (34–36). Human studies further suggest that HBOT reduces pro-inflammatory cytokine production during treatment, including IL-6 (37). These findings suggest that in tumor-associated immunosuppressive states, HBOT enhances the delivery and functionality of effector T cells after PD-1 blockade, enabling compatibility between peripheral tolerance and antitumor immunity. In autoimmune and chronic inflammatory contexts, however, HBOT shifts peripheral immunity back toward a tolerance-driven balance by downregulating pro-inflammatory pathways represented by IL-6 and IL-17 and strengthening the regulatory network mediated by FOXP3. This mechanism sets the stage for the subsequent remodeling of innate immune modules (**Figure 2**).

## 6 The role of innate-like lymphocytes and early immune phases in the reconstruction of PIT

Innate-like lymphocytes reside at the intersection of innate and adaptive immunity, and under the influence of HBOT, they are the first to sense changes in oxygen tension, translating metabolic and inflammatory signals into adjustments in early immune rhythms. In a study with healthy female volunteers receiving a single mild HBOT exposure at approximately 1.4 ATA, peripheral NK cell numbers and cytotoxic activity were significantly increased, while systemic inflammation remained stable. This indicates that, under conditions near immune homeostasis, low-dose HBOT preferentially enhances NK cell-mediated tumor and viral surveillance without incurring significant systemic inflammatory costs (19). In another study with healthy participants exposed to standard treatment pressure of approximately 2.4 ATA, a single HBOT exposure induced a transient increase in ROS, while overall antioxidant capacity increased, and plasma cytokine levels remained largely unchanged (38). Additionally, in healthy divers and endurance athletes, a single exposure to approximately 2.8 ATA of HBOT did not cause significant imbalances in oxidative stress or inflammation-related markers, suggesting the immunological safety of short-term exposure in healthy individuals (39). These data highlight that mild HBOT primarily enhances NK cell function with limited systemic oxidative stress effects, making it suitable for mild immune modulation and immune surveillance, while standard treatment pressures establish a new balance between controlled ROS increase and enhanced antioxidant defense, more closely resembling the actual immune resetting seen in pathological conditions. These findings indicate that immune effects may vary with different pressure protocols, but existing evidence is primarily derived from single exposure studies, and more prospective studies with comparable parameters are needed to define clear pressure thresholds or dose-response relationships.

**Figure 3:**
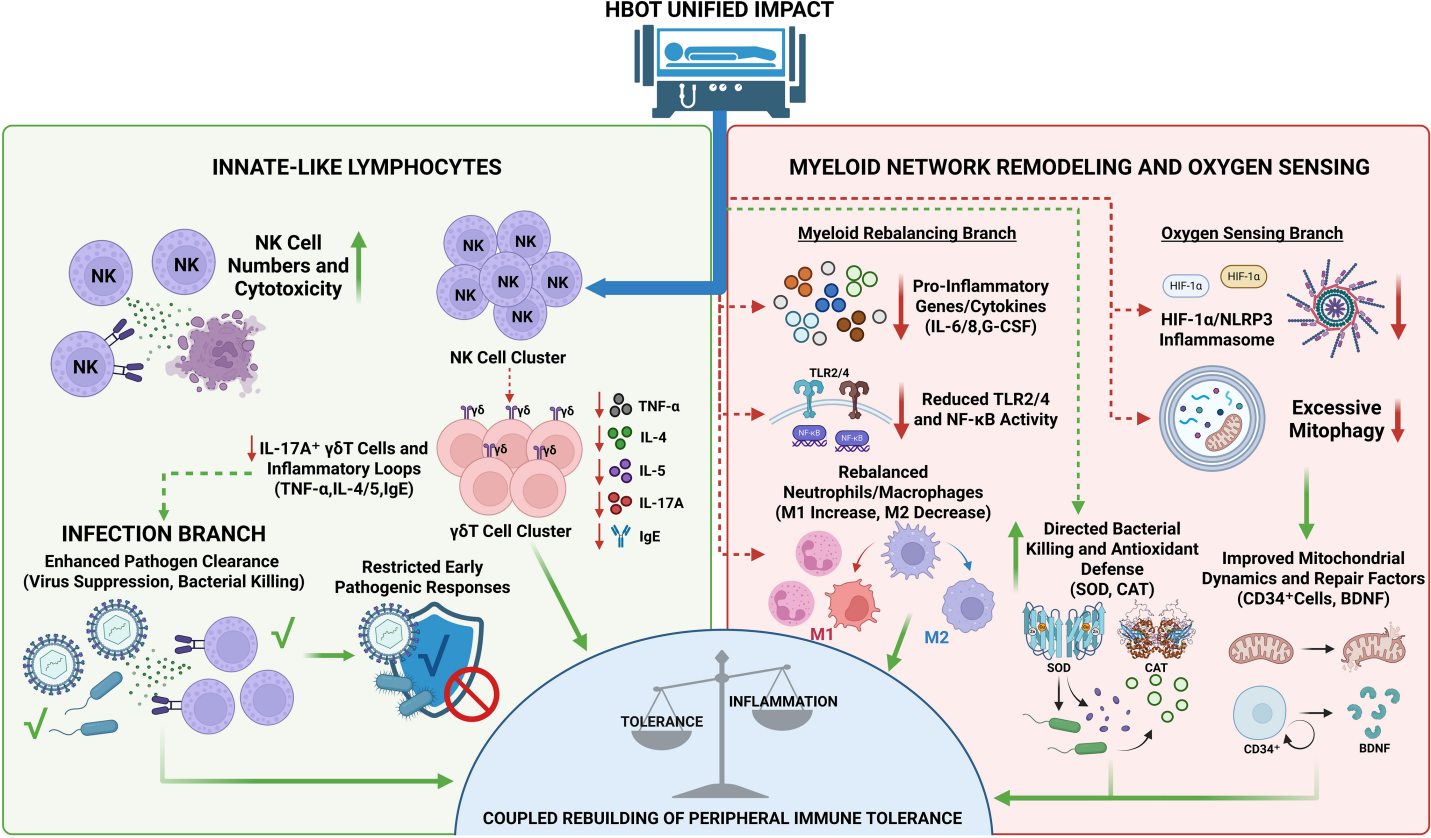
Hyperbaric oxygen therapy (HBOT)–driven remodeling of innate immunity and myeloid oxygen-sensing pathways.

In models of injury and localized inflammation, HBOT modulates γδ T cells and related inflammatory pathways in a manner that suppresses early pathogenic responses. In acute injury models of the central nervous system, such as spinal cord injury mice, HBOT at 2.0 ATA significantly reduced the proportion of IL-17A^+^ γδ T cells in spinal cord tissue, alleviated neutrophil infiltration, and improved motor function scores. This indicates effective disruption of the IL-17A-dependent γδ T cell amplification loop, thus mitigating secondary tissue damage (40). In studies of traumatic brain injury rats, HBOT not only reduced TNF-α and other inflammatory mediators in the brain but also reshaped the gut microbiome by increasing the abundance of Prevotella copri, transitioning the gut-brain axis from a high-inflammatory state toward a more homeostatic configuration (41). In early acute pancreatitis models, HBOT induced peripheral lymphocyte apoptosis and reduced pancreatic necrosis and disease severity (42). In a skin inflammation model, HBOT at 2.5 ATA transiently increased local ROS levels but significantly reduced IL-4, IL-5, and IgE levels, with a decrease in skin lesion scores, suggesting suppression of Th2-type inflammation in barrier tissues (43). From the central nervous system to peripheral tissues, and from IL-17A-driven γδ T cells to Th2-related cytokines, these findings collectively suggest that HBOT tends to dampen amplification circuits of inflammation in various tissues, promoting a quicker return to a state favorable for tolerance establishment (**Figure 3**).

In pathogen-associated and antiviral contexts, innate-like lymphocytes and mononuclear cells together form the frontline barrier for HBOT-mediated peripheral tolerance modulation. In an *in vitro* experiment with HIV-infected peripheral blood mononuclear cells (PBMCs), exposure to approximately 2.4 ATA of HBOT over several days significantly suppressed viral replication and reduced p24 antigen levels, with little effect on cell viability. This indicates that HBOT can reduce viral load without depleting immune cells (44). Combining this with earlier observations of enhanced NK cell cytotoxicity and only transient ROS elevation while maintaining overall stable systemic cytokines, it can be inferred that HBOT’s core feature in pathogen-related contexts is not simply to increase inflammation, but rather to enhance the efficiency of NK cells and mononuclear cells while avoiding a systemic shift to high-inflammatory states (19, 38). In this manner, HBOT may not only improve early control in chronic infections like viruses but also provide an environment with lower inflammatory noise and reduced tissue damage risk, potentially supporting long-term PIT mediated by FOXP3^+^ Tregs and memory T cells. This mechanism aligns with the previously discussed HBOT effects on effector T cell reprogramming in tumor and autoimmune settings.

This schematic summarizes the proposed effects of HBOT on innate immune remodeling across distinct physiological and pathological contexts. It integrates innate-like lymphocyte responses, myeloid network remodeling, and oxygen-sensing and mitochondrial pathways implicated in HBOT-associated immune rebalancing.

Additional symbols in **Figure 3**: Green dashed arrows indicate supportive/indirect positive associations. “↑/↓” denote upregulation/downregulation. The prohibition mark indicates restricted processes; the “✓” mark denotes beneficial outcomes/functional improvement; circular arrows indicate dynamic turnover or mobilization of repair-associated cells/factors. The balance icon indicates a net rebalancing toward homeostasis/tolerance. Symbols otherwise follow those defined in **Figure 2**.

## 7 Coupling and remodeling of myeloid network and oxygen sensing signals in PIT

### 7.1 Functional rebalancing of myeloid effector units

At the myeloid level, HBOT primarily resets the response thresholds of neutrophils and monocyte-derived macrophages by altering gene expression profiles and inflammatory mediator levels. Transcriptomic analysis of necrotizing soft tissue infection cohorts revealed that after treatment with approximately 2.8 ATA of HBOT, the expression of pro-inflammatory genes decreased, and pathways related to cellular homeostasis were modulated. These molecular-level changes were reflecting a broader attenuation of inflammatory and stress-response programs during treatment (45). Specifically, IL-6, IL-8, and G-CSF levels were significantly reduced during the acute phase, and lipid peroxidation markers such as LOOH and Malondialdehyde (MDA) were similarly decreased, indicating that HBOT does not simply reduce myeloid cell numbers, but instead shifts them from a high-inflammation, high-oxidative stress effector mode to a state of more controlled inflammation and reduced metabolic burden (46). Further research in multiple organ failure patients supported this, showing that HBOT at 2.5 ATA decreased peripheral TLR2 and TLR4 expression, reduced NF-κB activity, and improved organ function scores, suggesting that the TLR–NF-κB axis plays a key role in HBOT-mediated myeloid network rebalancing (47). A similar pattern of inflammation-related gene expression was observed in COVID-19 acute respiratory distress syndrome (ARDS) patients, where HBOT at 2.4 ATA not only improved PaO_2_ and alleviated hypoxia, but also reduced inflammation-related transcriptional signatures and improved clinical recovery, tightly linking improved oxygen delivery with reduced excessive myeloid activation (48). These findings indicate that HBOT has the capacity to reorganize the myeloid network in high-inflammatory states associated with infection, reducing the risk of systemic inflammatory storms while retaining essential antimicrobial functions—crucial for the re-establishment of PIT (**Figure 3**) (49).

Neutrophils, among the most oxygen-sensitive effector cells in the myeloid network, consistently demonstrate a robust evidence chain across clinical, animal, and *in vitro* studies. In patients with carbon monoxide poisoning, HBOT at approximately 2.0 ATA led to a decrease in CD18 expression on neutrophils and alleviated lipid peroxidation, indicating that adhesion activation and excessive oxidative bursts were controlled, reducing the risk of microvascular endothelial damage (50). This clinical observation was further validated in animal studies, where HBOT at 2.8 ATA reduced ICAM-1–dependent leukocyte aggregation, decreased myelin basic protein oxidation, and mitigated central nervous system demyelination (51). Subsequent *in vitro* experiments extended this logic to pathogen clearance, showing that neutrophils exposed to 2.0 ATA of HBOT exhibited enhanced bactericidal activity against Staphylococcus aureus, with synergy observed with antibiotics, while cell viability remained unchanged (52). HBOT also enhanced local oxygen penetration, reducing bacterial survival in Pseudomonas aeruginosa biofilms and improving the antibacterial efficacy of ciprofloxacin (53). Clinical, animal, and *in vitro* results collectively support the conclusion that HBOT enhances the directed bactericidal effect of neutrophils without exacerbating their collateral tissue damage, thus forming an infection model with lower inflammatory load and greater compatibility with PIT.

Monocyte-derived macrophages and dendritic cells play pivotal roles in HBOT-induced myeloid rebalancing, serving as critical bridges between innate and adaptive immunity. In ApoE-deficient atherosclerotic mice, HBOT at 2.4 ATA reduced the number of M1 macrophages in the aortic wall, decreased the expression of TNF-α and IL-6, and reduced plaque area, indicating that pro-inflammatory macrophages shifted toward a more reparative and tolerant lineage (54). In a glioblastoma model, HBOT at approximately 2.5 ATA decreased the proportion of M2 macrophages and increased the proportion of CD86^+^ M1 macrophages, restructuring the tumor microenvironment to promote T cell infiltration. This effect may act in concert with HBOT-associated macrophage repolarization toward a tumoricidal phenotype, supporting a tumor microenvironment more permissive for antitumor immunity (55). A systematic review of sepsis-related studies further supported this observation, where HBOT improved tissue oxygenation, reduced neutrophil adhesion, suppressed systemic inflammatory storms, and enhanced overall host homeostasis (56).

Collectively, the evidence from the myeloid network indicates that HBOT synchronously modulates neutrophils, monocyte-derived macrophages, and dendritic cells within a single framework, guiding peripheral immunity from a response mode dependent on high inflammation and tissue damage toward a balance that accommodates both pathogen clearance and tissue protection. This provides a solid cellular and functional basis for the subsequent mechanistic discussion on oxygen sensing and mitochondrial responses.

### 7.2 Translating oxygen sensing pathways and mitochondrial responses into tolerance

Changes in oxygen tension do not directly equate to immune suppression or activation; instead, they are translated into specific cellular activation states and remodeling signals through a series of oxygen-sensing and mitochondrial-related pathways. In the DSS colitis model, a molecular-level picture dominated by mucosal myeloid cells was observed under 2.0 ATA HBOT conditions. In this model, mRNA expression of HIF-1α and NF-κB-related inflammatory genes was significantly downregulated, while IL-1β and IL-6 levels decreased, and antioxidant enzyme activities, including superoxide dismutase (SOD) and catalase (CAT), increased (57). This combination of changes indicates that HBOT suppresses the persistent activation of hypoxia and classical inflammation transcription factors while enhancing antioxidant defense systems, transforming persistently elevated ROS into signals that can be buffered in time, thereby reducing the intensity of local inflammation cascades. In traumatic brain injury rat models, HBOT at 2.0 ATA suppressed NLRP3 inflammasome activation and its downstream Apoptosis-associated speck-like protein containing a CARD (ASC) and Caspase-1, reduced IL-1β and IL-18 expression in brain tissue, and decreased neuronal apoptosis, thus bringing neuroinflammation from an uncontrolled state back to a more manageable range (58). These findings suggest that HBOT, through coordinated regulation of HIF-1α–NF-κB and NLRP3 inflammasomes, integrates oxygen tension changes into an inflammation regulation mode that is more conducive to tolerance establishment (**Figure 3**).

Mitochondrial quality control pathways provide essential support for the above oxygen-sensing signals, maintaining cellular integrity and energy homeostasis. In carbon monoxide-induced lung injury rats, a single HBOT exposure at 2.5 ATA reduced excessive mitochondrial autophagy mediated by Pink1–Parkin, improved mitochondrial dynamics, and significantly alleviated lung tissue damage, indicating that maintaining appropriate mitochondrial renewal in a hyperoxic environment is a crucial prerequisite to prevent cells from entering energy crisis and necrotic inflammation (59). In *in vitro* experiments with human PBMCs, a single exposure to approximately 2.5 ATA induced DNA strand breaks but did not reduce cell viability. Instead, it enhanced antioxidant capacity, keeping the overall stress within a reversible range and resembling an immune preconditioning signal rather than irreversible damage (60). In long-term neuroimmune outcomes, patients with carbon monoxide poisoning and a risk of delayed encephalopathy showed an increase in peripheral CD34^+^ stem cell counts, elevated Brain-derived neurotrophic factor (BDNF) levels, and a reduced incidence of delayed encephalopathy after receiving HBOT at 2.0 ATA, demonstrating that HBOT promotes the mobilization of reparative progenitor cells and upregulates neurotrophic factors, helping the central immune and neural networks transition from a hyper-reactive state back to functional tolerance (61).

These oxygen-sensing and mitochondrial response pathways are coupled with the previously discussed myeloid effector cell rebalancing, and together with the two core mechanisms summarized earlier, they provide the metabolic and signaling foundation for HBOT’s role in establishing PIT. On one hand, HBOT regulates oxidative stress by coordinating ROS with antioxidants like SOD, while suppressing immune pathways. These effects, along with myeloid cell rebalancing, support immune tolerance. In NSTI, HBOT enhances myeloperoxidase (MPO), SOD, and heme oxygenase-1 (HO-1), with stronger effects in septic shock (62). On the other hand, by upregulating reparative factors like CD34^+^ progenitor cells and BDNF, and facilitating the reduction of IL-6 peaks and accelerated Treg expansion, HBOT ensures tighter coordination between tissue repair and immune tolerance programs over time (33). As a result, across multiple disease spectrums, HBOT not only alleviates local hypoxia and tissue damage but also completes the systemic reconstruction of PIT through multi-level interactions from molecular to cellular to systemic levels.

## 8 Discussion

This study explores the interaction between HBOT and PIT, systematically integrating 39 peer-reviewed studies. It incorporates evidence on how oxygen tension, immune metabolism, and the regulatory axis of PIT interact to modulate immune responses. The study aims to elucidate how HBOT, through oxygen tension-driven immune metabolic reprogramming, alters the thresholds of PIT, thereby recalibrating the balance between immune effector functions and immune tolerance in different disease contexts. It is important to note that the included studies show considerable heterogeneity in exposure protocols and endpoint assessments, meaning that related inferences should be interpreted in the context of specific study conditions and observed indicators. Animal studies often involve prolonged treatment courses (multiple days or weeks), allowing for the observation of immune lineage and functional state remodeling over multiple time points. Human studies, however, typically involve single exposures, primarily capturing short-term ROS dynamics and transient fluctuations in peripheral cell subsets. *In vitro* studies, by eliminating the regulatory background of the organism, primarily reflect the direct effects of acute hyperoxic stress on cellular effector functions and stress markers. Based on this structure of evidence, the more robust conclusions regarding Treg/Th17 shifts rely on multiple treatment course studies, while the threshold of different pressure protocols and dose-response relationships remain exploratory and need further verification in prospective studies with comparable parameters.

In high-inflammatory, glycolysis-biased contexts, such as autoimmune diseases and chronic inflammatory conditions, HBOT typically results in a reduction of pro-inflammatory cytokine levels, an increase in FOXP3^+^ Treg proportions, and a shift of the Treg/Th17 ratio back toward physiological levels. This suggests a partial recalibration of PIT thresholds, which have been disrupted due to inflammation load (63, 64). From the perspective of cellular metabolism and subcellular structures, HBOT alleviates tissue hypoxia and excessive oxidative stress, weakens the glycolysis bias driven by HIF-1α, and reduces the dependency of effector T cells and Th17 cells on high glycolysis. At the same time, HBOT improves mitochondrial function and ROS homeostasis, facilitating a metabolic profile in Tregs that is dominated by fatty acid oxidation and OXPHOS (65). These changes in the metabolic environment further impact transcriptional regulatory programs sensitive to metabolism, enhancing Treg-related gene expression while limiting the expansion of pro-inflammatory lineages, thereby strengthening key components of regulatory suppression and functional unresponsiveness within the PIT network (66, 67). Thus, in high-inflammatory settings, HBOT does not merely suppress immune responses but instead lowers the metabolic thresholds for pathological effector responses and upregulates the activity of tolerance-related modules, pushing PIT back toward a more homeostatic set point.

In addition to these direct immunometabolic effects, a gut-mediated component may also contribute to immune rebalancing in high-inflammatory states. Intestinal oxygen and redox tone are established ecological constraints that can reshape microbial niches and metabolite availability and thereby influence dysbiosis-linked inflammatory outputs (68, 69). Against this backdrop, HBOT-induced shifts in tissue oxygenation under inflammatory conditions could plausibly propagate to the gut microenvironment, with secondary effects on microbiota-derived immunoregulatory cues that interface with the Treg and Th17 axis discussed above. Early evidence from distinct indications is compatible with this possibility. In Crohn’s disease, HBOT was accompanied by clinical improvement together with coordinated changes in microbial community features, and stool transfer from post-HBOT donors mitigated inflammation in recipient mice, supporting a contributory microbial component (70). Similarly, in a traumatic brain injury rat model, HBOT increased Prevotella copri and coincided with reduced TNF-α and attenuated neuroinflammatory readouts (41). Nevertheless, current evidence remains limited in scope and heterogeneous across indications, and establishing definitive causality in humans will require well-designed longitudinal studies.

In deeply hypoxic and metabolically suppressed lesions, such as malignant tumors and certain chronic infections, this regulatory axis exhibits opposing effects depending on the immune and metabolic background. These lesions are typically characterized by a significant drop in local tissue oxygen tension, the accumulation of lactate and other metabolic inhibitors, impaired antigen-presenting cell function, and the development of exhausted phenotypes in CD8^+^ T cells and NK cells. This local immune environment essentially represents a pathological “over-tolerant” state (71). In such contexts, HBOT increases local oxygenation levels, alleviates the lactate load, and improves mitochondrial metabolism, which helps restore the ability of antigen-presenting cells to process and present antigens. Additionally, it increases the infiltration and effector functions of CD8^+^ T cells and NK cells (72). Under conditions of improved ROS signaling and mitochondrial quality control, effector T cells are more likely to transition from a state of deep exhaustion to partial functional recovery or memory lineage differentiation (73). At the nuclear level, the transcriptional programs associated with co-inhibitory receptor-driven exhaustion are partially downregulated, accompanied by a moderate lowering of local PIT thresholds, thus alleviating the pathological tolerance in the lesions (74). However, the current research lacks sufficient evaluation of the systemic costs of self-tolerance, and due to limitations in sample size and follow-up duration, safety-related inferences must be made cautiously. This group of findings suggests that in tumor and chronic infection contexts, HBOT is more aligned with relieving metabolic suppression, selectively weakening the excessively high tolerance barriers within the lesions, thereby enhancing effective immune responses against tumors or pathogens (75).

**Figure 4:**
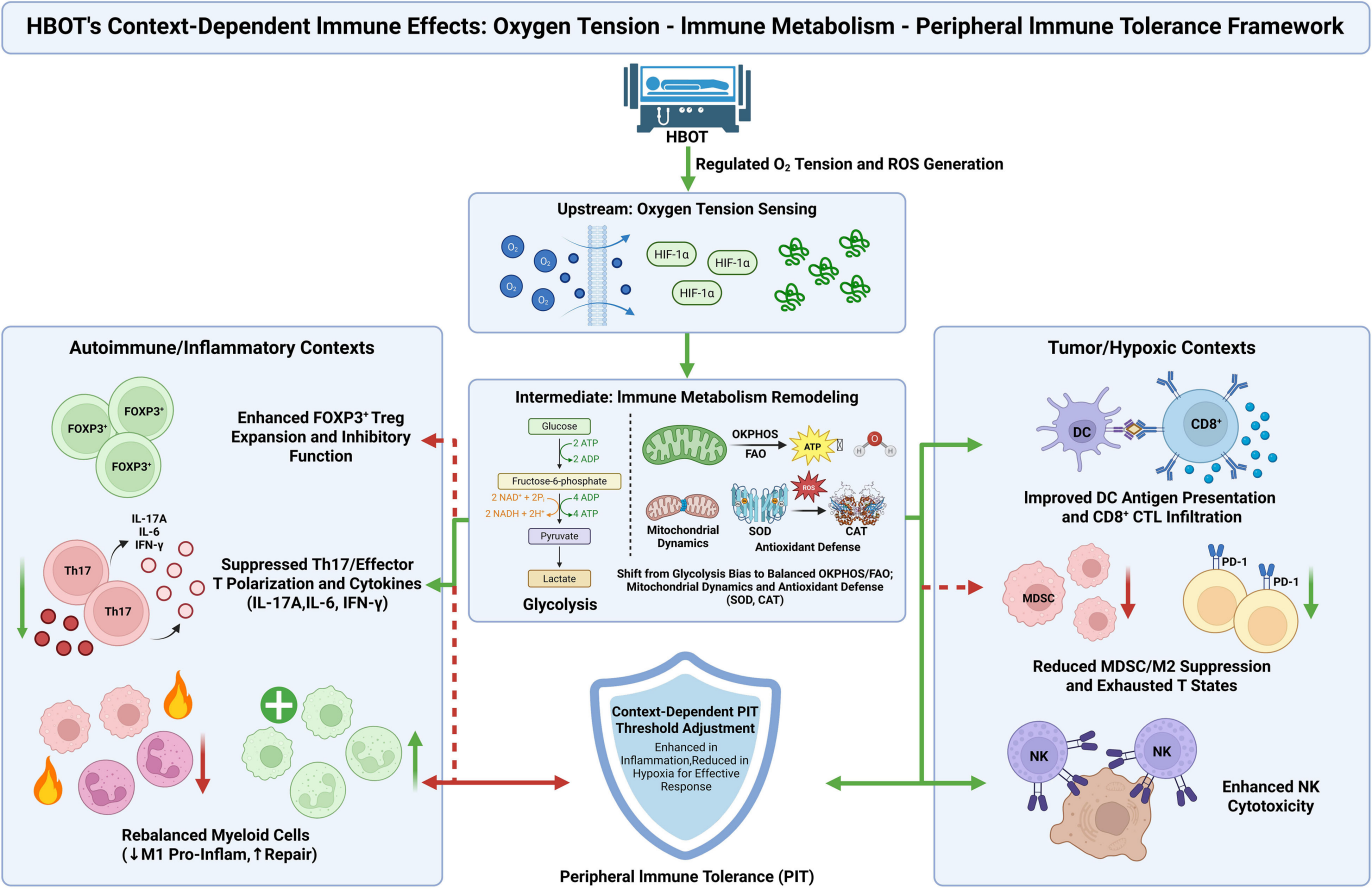
The oxygen tension–immunometabolism–peripheral immune tolerance (PIT) framework of hyperbaric oxygen therapy (HBOT).

In populations close to immune homeostasis or with non-inflammatory indications, existing limited follow-up data have not suggested that HBOT induces sustained peripheral immune dysregulation or tolerance disruption (76). This observation resonates with the two previously discussed disease contexts, suggesting that the effect of HBOT on PIT can be uniformly explained within the same regulatory axis. When baseline inflammation load is low and tissue oxygenation is close to physiological levels, the immune cell metabolism and PIT network are already near a steady state. In such cases, the changes in oxygen tension induced by HBOT cause only limited recalibration at the immune metabolic level, and consequently, the impact on PIT thresholds is relatively small. Combining the three scenarios discussed above, a unified explanatory framework can be developed (**Figure 4**). HBOT influences PIT through regulating upstream oxygen tension, inducing immune metabolic remodeling at the cytoplasmic and mitochondrial levels, and altering gene expression and regulatory programs related to PIT at the nuclear transcriptional level. In autoimmune and chronic inflammatory diseases, HBOT primarily enhances tolerance-related pathways, while in tumors and chronic infections, it reduces excessive tolerance in the lesion sites. In populations with immune states close to homeostasis, the overall impact of HBOT is limited. This differential pattern of immune modulation is mainly related to baseline inflammation load, tissue hypoxia, and metabolic status.

This schematic outlines a conceptual framework linking HBOT associated shifts in oxygen tension to immunometabolic remodeling and PIT. It integrates the oxygen tension to metabolism axis with context dependent recalibration of PIT across autoimmune or chronic inflammatory conditions and hypoxic tumor microenvironments.

Additional symbols in **Figure 4**: “+” indicates an increase or improvement of the indicated process/outcome. The shield icon denotes context-dependent adjustment of the PIT threshold. Bidirectional arrows indicate reciprocal interactions between connected modules; green vs red denotes the dominant direction of the net effect (enhancement vs suppression). This differs from **Figure 2**, where the green bidirectional arrow is used specifically to denote immune-balance remodeling. Other symbols follow those defined in **Figure 2** and **3**.

Based on this unified framework, the effect of HBOT on PIT can be clearly summarized as a continuous regulatory process that involves upstream oxygen tension sensing, intermediate immune metabolic remodeling, and downstream immune cell lineage and functional state adjustments. Oxygen tension changes mainly regulate ROS generation and antioxidant defense systems through HIFs and related oxygen-dependent pathways (77, 78). These pathways shape the metabolic environment of immune cells, subsequently affecting the balance between glycolysis and OXPHOS in the cytoplasm and mitochondria, the redistribution of fatty acid oxidation and glutamine metabolism, and mitochondrial dynamics and autophagy processes. Collectively, these metabolic and lineage changes dictate the plasticity of T cells and myeloid cells in differentiating toward effector, regulatory, memory, and exhausted functional states (79). These metabolic and lineage shifts are then integrated through nuclear transcription programs, resulting in coordinated changes in key immune mechanisms, such as the Treg/Th17 balance, effector T cell exhaustion, memory lineage switching, and polarization of key myeloid subpopulations. Ultimately, these changes manifest as an upward or downward shift in the PIT threshold (80, 81). In this sense, HBOT is better understood as an intervention tool that modulates oxygen tension, immune metabolism, and PIT, rather than a simple immune stimulator or immunosuppressant.

The findings of this study have significant theoretical implications and potential translational value. HBOT can be viewed not only as a traditional physical adjuvant therapy but also as an immune-metabolic intervention that modulates PIT thresholds. Future research should shift from an overall evaluation of therapeutic efficacy to defining appropriate patient populations and conditions of action. Specifically, future studies should clarify which oxygen tension trajectories and exposure models can regulate PIT thresholds to more favorable ranges for disease control under different immune and metabolic backgrounds. Existing evidence suggests that future research should avoid focusing solely on individual inflammatory markers or specific cell ratios and instead adopt PIT as the central framework. This framework should incorporate composite endpoints, including the Treg/Th17 axis, effector T cell exhaustion and memory lineage, myeloid cell subsets, ROS levels, and mitochondrial function, while employing multi-timepoint evaluation strategies to assess the dynamic time characteristics of the transition from innate to adaptive immune remodeling. Current results indicate that patients with significant Treg/Th17 imbalance and local hypoxia in autoimmune and inflammatory diseases may be better suited for HBOT intervention. In the field of cancer immunotherapy, HBOT could be prioritized as a potential adjunct to immune checkpoint inhibitors, adoptive cell therapy, or vaccines, particularly in tumor types where hypoxia drives immune evasion, and should be evaluated in related clinical studies.

However, translating the regulatory axis proposed in this study from a conceptual framework to evidence-based conclusions still faces multiple limitations. The existing studies exhibit high heterogeneity in terms of disease spectra, HBOT pressure and treatment parameters, immune endpoint selection, and other factors. Furthermore, many studies have small sample sizes, and key information related to randomization, blinding, and loss to follow-up is often inadequately reported, making bias risks better suited for qualitative rather than quantitative assessment. Given these realities, this study did not apply a unified bias risk evaluation scale to all included studies, nor was it pre-registered on platforms like PROSPERO. Instead, we relied on predefined inclusion and exclusion criteria and conducted a narrative analysis of core methodological elements and their limitations in each mechanism section, thus delimiting the evidence base and applicability of the mechanistic inferences made in this study. To capture mild HBOT-related evidence as comprehensively as possible, this study lowered the pressure threshold to 1.4 ATA; however, only one study using approximately 1.4 ATA met the criteria (19). The increase in tissue oxygen tension and cumulative oxygen load in this study is difficult to directly compare with conventional protocols using ≥ 2.0 ATA, and it is more appropriate as an exploratory observation for mild hyperbaric exposure rather than a reliable basis for dose-response relationships. Moreover, most studies employed limited time points for measurements, which are insufficient to support rigorous time-series analysis of the presumed causal pathways. Prospective randomized controlled trials focused on PIT restoration or immunosenescence reversal as primary outcomes remain scarce in several key disease areas, and high-level evidence to support these conclusions is lacking.

Given these limitations, future research needs to adopt more targeted designs at the basic, translational, and clinical levels. At the basic and translational levels, studies using representative autoimmune diseases, inflammatory storms, and tumor models, with consistent or comparable HBOT parameters, could integrate multi-timepoint single-cell omics and metabolomics techniques. Such studies should focus on the hypothesized regulatory sequence driven by oxygen tension changes, which alters ROS dynamics, reprograms metabolic pathways, and ultimately affects the Treg/Th17 axis and effector T cell lineage remodeling. This approach could identify key nodes with potential regulatory significance. At the clinical level, it is recommended that cohort studies and randomized controlled trials predefine relatively uniform PIT endpoints and integrate oxygen tension change curves, dose and exposure parameters, and clinical outcomes into the same multivariable analysis models to define potential immune modulation treatment windows in different disease types and immune baseline states. Furthermore, the combined application of HBOT with other immune-metabolic or tolerance-regulating approaches should be further evaluated to explore whether the concept of oxygen tension, immune metabolism, and PIT can be translated into a feasible, individualized intervention strategy.

Overall, the regulatory axis centered around oxygen tension, immune metabolism, and PIT proposed in this study provides a relatively structured and testable theoretical framework for integrating the complex immunological effects of HBOT in various disease contexts. Despite existing limitations in the scope and quality of evidence, the conclusions drawn still need further validation through more rigorous basic and clinical studies. However, systematically researching the concept of PIT remodeling through immune-metabolic regulation holds promise for clarifying the immunomodulatory effects of HBOT in autoimmune diseases, inflammatory storms, cancer immunotherapy, transplantation, and other fields, and evaluating its potential clinical translation value.

## Data Availability

The original contributions presented in the study are included in the article/supplementary material, further inquiries can be directed to the corresponding author/s.
